# The Effects of Anchor Schemes on Performance Fatigability, Neuromuscular Responses and the Perceived Sensations That Contributed to Task Termination

**DOI:** 10.3390/jfmk8020049

**Published:** 2023-04-25

**Authors:** Robert W. Smith, Terry J. Housh, Jocelyn E. Arnett, John Paul V. Anders, Tyler J. Neltner, Dolores G. Ortega, Richard J. Schmidt, Glen O. Johnson

**Affiliations:** 1 Exercise Physiology Laboratory, Department of Nutrition and Health Sciences, University of Nebraska—Lincoln, Lincoln, NE 68510, USA; bsmith80@huskers.unl.edu (R.W.S.);; 2 The Exercise Science Program, Department of Human Sciences, The Ohio State University, Columbus, OH 43017, USA

**Keywords:** fatigue, torque, perception, exertion, anchor, electromyography

## Abstract

The present study examined the effect of anchor schemes on the time to task failure (TTF), performance fatigability, neuromuscular responses, and the perceived sensations that contributed to task termination following the sustained, isometric forearm flexion tasks. Eight women completed sustained, isometric forearm flexion tasks anchored to RPE = 8 (RPEFT) and the torque (TRQFT) that corresponded to RPE = 8. The subjects performed pre-test and post-test maximal isometric contractions to quantify performance fatigability and changes in electromyographic amplitude (EMG AMP) and neuromuscular efficiency (NME). In addition, the subjects completed a post-test questionnaire (PTQ) to quantify the contributions of perceived sensations to task termination. Repeated measure ANOVAs were used to assess the mean differences for TTF, performance fatigability, and neuromuscular responses. Wilcoxon Signed Rank Tests were used to assess the differences between anchor schemes for the average values from the PTQ item scores. For TTF, the RPEFT was longer than the TRQFT (174.9 ± 85.6 vs. 65.6 ± 68.0 s; *p* = 0.006). Collapsed across the anchor scheme, there were decreases in torque (23.7 ± 5.5 Nm vs. 19.6 ± 4.9 Nm; *p* < 0.001) and NME (1.00 ± 0.00 vs. 0.76 ± 0.15; *p* = 0.003). There were no significant (*p >* 0.577) changes for EMG AMP. For the PTQ, there were no differences (*p >* 0.05) between anchor schemes. There were, however, inter-individual differences in the response scores. The current findings indicated that performance fatigability was likely due to peripheral fatigue (based on NME), not central fatigue (based on EMG AMP). Furthermore, the use of a PTQ may serve as a simple tool to assess the contributions of perceived sensations to task termination.

## 1. Introduction

The study of fatigue has become increasingly specialized with a variety of definitions and divisions in research outcomes. The multiple definitions of fatigue have complicated the ability to characterize performance-related and/or psychophysiological responses during a fatiguing task. Therefore, Kluger et al. [1] and Enoka and Duchateau [2] proposed holistic, unified taxonomies of fatigue that included both performance (performance fatigability) and perceived (perceived fatigability) aspects of fatigue, which may act independently or in conjunction with one another. More recently, Behrens et al. [3] provided an updated framework of the taxonomies of fatigue described by Kluger et al. [1] and Enoka and Duchateau [2], and they proposed to define performance fatigability as a quantifiable decrease in maximal voluntary torque production, which can be informed by central and/or peripheral factors. These central and peripheral factors are manifested as physiological and mechanical perturbations throughout the nervous system and within the muscle, resulting in a reduced capacity to maximally generate torque. Behrens et al. [3] stated that the central factors include aspects related to muscle activation, such as changes in voluntary activation, excitability of cortical motoneurons and spinal α-motoneurons, afferent feedback, and/or neuromuscular propagation. Additionally, Behrens et al. [3] provided an updated framework that indicated that performance fatigability can be affected by peripheral factors that are associated with contractile function, such as reduced blood flow, metabolite accumulation, disruptions to sarcolemma excitability, Ca^2+^ kinetics, excitation–contraction coupling, and the tension-generating capacity of the cross-bridges. Perceived fatigability refers to an increase in the subjective perception of fatigue that emerges during a sustained task and is affected by factors related to the psychophysiological state of the individual, including perceptions of effort, pain, or discomfort, as well as motivation, affective valence (i.e., general indication of how a person currently feels), mood, expectations, and/or executive function (i.e., higher-level cognitive skills involved with control and coordination of cognitive abilities and behaviors) [1,2,3].

It has been suggested [4] that the regulation of exercise intensity and the decision to terminate the task are the result of the “…interpretation of the momentary rating of perceived exertion (RPE)” (p. 821). According to Robertson et al. [5], RPE serves as a general indicator of the perception of exertion, which includes components such as strain, discomfort, and/or fatigue experienced during the task. Previously, Tucker [6] proposed the RPE-Clamp model, which describes how exercise intensity is regulated when it is anchored to a constant RPE. The RPE-Clamp model [6] suggests that when intensity is anchored to a constant RPE value, the initial exercise intensity is set in an anticipatory manner based on previous experiences, training status, and the expected duration of the task. Furthermore, the initial exercise intensity is perceived by the individual as matching the RPE that they are anchoring to. When exercise is sustained, fatigue-induced physiological changes and afferent feedback from systems that are directly and indirectly involved with the exercise task are interpreted within the brain and result in continuous adjustments to the exercise intensity to maintain the prescribed RPE.

Given the unique nature of the RPE-Clamp model, recent studies have examined the interactions among factors associated with performance fatigability and perceived fatigability, during [7,8,9] and following [10,11] sustained, isometric tasks that are anchored to a high perceptual intensity. For example, Arnett et al. [10] reported mean decreases in the torque and amplitude of the electromyographic signal (EMG AMP) following a sustained, isometric forearm flexion task anchored to RPE = 8. It was hypothesized that the fatigue-induced changes in torque may have been due to a combination of central and peripheral factors, as evidenced by the mean decreases in EMG AMP. Furthermore, Smith et al. [12] reported mean decreases in the torque and EMG AMP during a sustained, forearm flexion task anchored to RPE = 7, and hypothesized that the subjects may have voluntarily reduced the torque due to afferent feedback as a result of peripheral fatigue (based on EMG AMP) or increased perceived fatigability (i.e., loss of motivation to continue). Thus, by applying the RPE-Clamp model [6], the central and peripheral factors related to performance fatigability, as well as the psychophysiological factors associated with perceived fatigability, can be examined simultaneously during and following sustained, isometric tasks anchored to a constant RPE. Only a single study [13], however, has compared the changes in force and neuromuscular parameters following a task anchored to a constant RPE versus those from a task anchored to the force produced at the initiation of the RPE task. Specifically, Keller et al. [13] reported no differences between anchor schemes (anchored to a constant RPE versus anchored to a constant torque) for the time to task failure (TTF), EMG AMP, or performance fatigability during bilateral, isometric leg extensions. Whether or not the anchor scheme influences the torque and neuromuscular responses during upper body and unilateral tasks remains to be elucidated.

Exercise-induced adjustments to the neuromuscular system are often assessed by examining the time domain of the EMG signal. It has been suggested [14,15] that EMG AMP represents muscle excitation attributed to motor unit recruitment, firing rate, and/or synchronization. For example, previous studies have utilized EMG AMP to make inferences regarding fatigue-induced adjustments in motor unit activation strategies during and after sustained, isometric forearm flexion [7,8,9,10] and leg extension [11,16,17] tasks, with torque or force anchored to a constant RPE using the OMNI-RES (0–10) scale [18,19,20]. In addition, previous studies [21,22,23] have examined the ratio between normalized torque (or force) and normalized EMG AMP to estimate neuromuscular efficiency (NME: a measure of the level of excitation required to generate a given amount of torque). It has been suggested [22] that NME is characterized as peripheral fatigue and NME may be influenced by a buildup of metabolites that results in excitation–contraction coupling failure. Thus, neuromuscular parameters such as EMG AMP and NME may be useful for investigating fatigue-induced changes following a sustained, isometric task.

While a number of studies [6,24,25,26] have provided hypotheses regarding the underlying mechanisms involved with the regulation of exercise intensity and, ultimately, task failure, few have described these processes within the context of performance fatigability and perceived fatigability. The Integrative Governor Theory [27] hypothesizes that the regulation of exercise intensity, development of fatigue, and the outcome of a sustained task are based on a continuous and dynamic competition between the central and peripheral factors that are associated with performance fatigability, as well as the psychophysiological factors that are associated with perceived fatigability based on metabolic setpoints and the maintenance of homeostasis via negative feedback loops that prevent the individual from reaching a catastrophic level. This model, however, does not address specific psychophysiological factors [3,28] that are reportedly associated with perceived fatigability, such as overall perceived fatigue [29], perceptions of effort, pain, and discomfort [29,30,31,32], affective valence [29], and motivation [33]. Furthermore, previous studies [25,34,35,36,37] have attempted to provide a theoretical explanation for the regulation of exercise intensity and the decision to terminate exercise during a task that is anchored to a constant relative intensity or over the course of a known distance or duration. No studies, however, have sought to compare the perceived factors that contribute to the decision to terminate a fatiguing task when the tasks are anchored to a constant RPE or the torque produced at the initiation of the RPE task. Therefore, the purpose of the present study was to examine the effect of anchor schemes on TTF, performance fatigability, neuromuscular responses, and the perceived sensations that contributed to task termination following sustained, isometric forearm flexion tasks. Based on the findings of previous studies [3,13,22,31,32,38], it was hypothesized that (1) TTF would be greater for the RPE fatigue task (RPEFT) versus the torque fatigue task (TRQFT) [13]; (2) there would be no differences between anchor schemes for the fatigue-induced changes in performance fatigability or neuromuscular responses [13]; (3) perceived fatigability of the muscles involved with the forearm flexion tasks would be greater for the TRQFT versus the RPEFT; and (4) the psychological factors related to perceived fatigability would have a greater level of contribution to the decision to terminate the task for the RPEFT than the TRQFT [38,39].

## 2. Materials and Methods

### 2.1. Subjects 

An a priori G*Power3 analysis determined that a minimum of 6 subjects were required to demonstrate mean differences between 2 dependent groups using repeated-measures ANOVAs, based on an effect size of $\eta_{p}^{2}$ = 0.551 [40], a power of 0.95, and an alpha of 0.05. Thus, eight recreationally active [41] women (mean ± SD: age = 21.0 ± 3.2 years; height = 168.3 ± 8.0 cm; body mass = 68.3 ± 8.1 kg; aerobic exercise = 2.1 ± 1.6 h; anaerobic exercise = 5.7 ± 3.7 h) with no known cardiovascular, metabolic, or muscular diseases volunteered to participate in this study. The subjects in the present study were part of a larger, multiple independent and dependent variable investigation, but none of the data in the current study have been previously published [10]. The subjects visited the laboratory for an orientation session and two testing visits separated by at least 24 h, and all testing was scheduled at approximately the same time of day. In addition, the subjects were instructed to avoid upper body exercise for at least 24 h prior to testing and avoid consumption of caffeine for at least 6 h prior to testing. 

### 2.2. Ethical Approval

The study was approved by the University of Nebraska–Lincoln Institutional Review Board for Human Subjects (IRB Approval #: 20201220785FB; 23 November 2021), and all subjects completed a Health History Questionnaire and signed a written informed consent prior to testing.

### 2.3. Time Course of Procedures

The subjects visited the laboratory on three separate occasions (orientation session, test visit 1, and test visit 2) separated by 24 to 96 h. The initial visit was an orientation session where demographic information was recorded, and the subjects were familiarized with the standardized warm-up, the testing protocol, and the Omnibus-Resistance Exercise 0–10 (OMNI-RES) [18,19,20] anchoring procedures were read to them (Table 1; Appendix A). Test visit 1 included the standardized warm-up, followed by the anchoring procedures, which included 2 repetitions of 3 s pre-test forearm flexion maximal voluntary isometric contractions (MVICs) (~10 s of rest between MVIC attempts) to set a perceptual anchor to RPE = 10. This was followed by a sustained, isometric forearm flexion task anchored to RPE = 8 to task failure. The subjects then performed 2 repetitions of 3 s post-test forearm flexion MVICs in a manner that was identical to the pre-test MVICs. Finally, the subjects completed a post-test questionnaire that included a brief, open-ended response question and five separate items that were rated on a 6-point scale to determine the contribution and magnitude of the items related to the subject’s decision to discontinue the task. Test visit 2 included the standardized warm-up, along with 2 repetitions of 3 s pre-test forearm flexion MVICs (~10 s of rest between MVIC attempts) followed by a sustained, isometric forearm flexion task anchored to the torque that was produced during the first 1 s of the RPE = 8 task from test visit 1. The subjects then performed 2 repetitions of 3 s post-test forearm flexion MVICs and completed the post-test questionnaire. All forearm flexion contractions were performed at an elbow joint angle of 125° (EJ_125_) to reflect the point in the range of motion that approximated maximal isometric torque production [42].

### 2.4. Orientation Session

During the orientation session, the dominant arm (based on throwing preference), age, height, and body mass of the subjects were recorded. In addition, the subjects were oriented to their testing position on the upper body exercise table (UBXT) of the calibrated isokinetic dynamometer (Cybex II, Cybex International Inc., Medway, MA, USA) with the lateral epicondyle of the humerus of the dominant arm aligned with the lever arm of the dynamometer at EJ_125_. The subjects were then familiarized to the OMNI-RES scale [18,19,20] and read the standardized OMNI-RES instructions [12,43] (Appendix A). The subjects then completed the standardized warm-up consisting of 4 repetitions of 3 s submaximal (~50–75% of their maximal effort), isometric forearm flexion contractions at EJ_125_ as well as 2 repetitions of 3 s isometric forearm flexion maximal voluntary isometric contractions (MVICs) at EJ_125_ to set a perceptual anchor corresponding to RPE = 10. Finally, the subjects performed a brief (approximately 1 min), sustained, isometric task anchored to RPE = 8 at EJ_125_ to become familiarized with the testing and anchoring procedures. Finally, the subjects were familiarized to the post-test questionnaire (PTQ) and read the standardized PTQ instructions (Appendix B).

### 2.5. Test Visits

During test visit 1, the subjects were positioned in accordance with the Cybex II user’s manual on the UBXT with the lateral epicondyle of the humerus of the dominant arm aligned with the lever arm of the dynamometer at EJ_125_. Once positioned, the subjects performed the standardized warm-up (Table 1), followed by 1 min of rest. After the warm-up, the subjects were again read the OMNI-RES instructions relating to the anchoring procedures. The subjects then performed 2 repetitions of 3 s of forearm flexion MVICs on the dynamometer at EJ_125_. The MVICs also served to remind the subjects of the perceptual anchor corresponding to RPE = 10. Following the MVIC trials, the sustained, isometric forearm flexion task anchored to RPE = 8 (OMNI-RES scale) was performed at EJ_125_ (RPEFT). During the sustained isometric task, the subjects were unaware of torque and elapsed time to avoid pacing strategies [44]. During the RPEFT, the subjects were free to adjust torque to maintain the prescribed RPE = 8, and task failure was defined as the time when torque was reduced to zero. In addition, during the RPEFT, the subjects were reminded to be attentive to sensations such as strain, intensity, discomfort, and fatigue that were felt during the task to maintain the appropriate level of exertion [20,39]. Furthermore, the subjects were continuously reminded that there were no incorrect contractions or perceptions and were reminded to relate levels of exertion to the previously set anchors of RPE = 0 and RPE = 10. Throughout the RPEFT, the subjects were asked their RPE every 30 s to assure compliance with the prescribed RPE = 8. Upon task failure, the RPEFT was terminated and the TTF was recorded. Immediately after task failure, 2 repetitions of 3 s post-test MVICs were performed in a manner identical to the pre-test MVICs, and the PTQ was completed. After test visit 1 was concluded, the torque produced during the first 1 s of the RPEFT was recorded.

During test visit 2, the positioning on the UBXT and arm alignment for the subjects were identical to test visit 1. Once positioned, the subjects performed the standardized warm-up (Table 1), followed by 1 min of rest. After the warm-up, the subjects performed 2 repetitions of 3 s of forearm flexion MVICs on the calibrated dynamometer at EJ_125_. Following the MVIC trials, the subjects performed a sustained, isometric forearm flexion task anchored to the torque (TRQFT) produced during the first 1 s of the RPEFT. This was conducted so that both fatiguing tasks began at the same initial torque value. During the TRQFT, the target torque was displayed on a computer screen to allow the subjects to track their torque output throughout the sustained, isometric task. The TRQFT was sustained to task failure, which was defined as the time point at which the subjects could no longer maintain the target torque despite strong verbal encouragement. Upon task failure, the task was terminated and the TTF was recorded. Immediately after task failure, 2 repetitions of 3 s post-test MVICs were performed in a manner identical to the pre-test MVICs, and the PTQ was completed. Strong verbal encouragement was provided for all MVIC trials.

### 2.6. Post-Test Questionnaire

The PTQ (Appendix B) was provided to the subjects following the completion of the post-test MVIC trials to identify the location, contribution, and magnitude of perceived sensations and psychological factors that subjects perceived contributed to the subject’s decision to discontinue the tasks. The subjects were again read the standardized PTQ instructions after completion of the RPEFT and TRQFT. In addition, the PTQ included five separate items that were rated on a 6-point (0–5) scale. A rating of 0 indicated that the item had no contribution to the decision to terminate the task, and a rating of 5 indicated that the item had the greatest level of contribution out of the five items to the decision to terminate the task. Ratings of 1–4 indicated the varying levels of intensity of contribution related to the decision to terminate the task (i.e., a rating of 5 indicated a greater level of contribution to the decision to terminate the task than a rating of 4, a rating of 4 indicated a greater level of contribution to the decision to terminate the task than a rating of 3, and so on). Furthermore, items that were rated equally indicated the same level of intensity in their contribution to the decision to terminate the task. The five items included: biceps brachii (BB), forearm muscles (FM), hand muscles (HM), loss of motivation, and loss of focus. The primary and synergistic muscle groups associated with forearm flexion (i.e., BB, FM, and HM) were selected to identify the potential locations of sensations such as strain, discomfort, and/or pain that occurred during the task. Loss of motivation was selected to identify if the task, for whatever reason, became sufficiently unattractive to the subject and resulted in a decision to terminate the task. Finally, loss of focus was selected to identify if focus (i.e., attention) shifted to other tasks or responsibilities, which may have caused a disruption in the ability to maintain the prescribed RPE and/or torque value.

### 2.7. Electromyographic and Torque Acquisition

During the testing visit, bipolar (30 mm center-to-center) EMG electrodes (pregelled Ag/AgCl, AccuSensor; Lynn Medical, Wixom, MI, USA) were attached to the BB of the dominant arm based on the recommendations of the Surface Electromyography for the Non-Invasive Assessment of Muscles [45]. A reference electrode was also placed on the styloid process of the radius of the forearm. Prior to electrode placement, the skin was shaved, carefully abraded, and cleaned with alcohol. The electrodes were placed over the BB between the medial acromion and the antecubital fossa, at one-third the distance from the antecubital fossa. The raw EMG signal was digitized at 2000 Hz with a 12-bit analog-to-digital converter (Model MP150; Biopac Systems, Inc., Goleta, CA, USA) and stored on a personal computer (Acer Aspire TC-895-UA91 Acer Inc., San Jose, CA, USA) for analyses. The EMG signal was amplified (gain: ×1000) using a differential amplifier (EMG2-R Bionomadix, Biopac Systems, Inc., Goleta, CA, USA; bandwidth—10–500 Hz) and digitally bandpass filtered (fourth-order Butterworth) at 10–500 Hz. Signal processing was performed using custom programs written with LabVIEW programming software (version 20.0f1, National Instruments, Austin, TX, USA). A 1 s epoch from the center of the 3 s of forearm flexion MVIC with the greatest torque production was used to calculate the AMP (root mean square) for the EMG (µVrms) signal. The torque signals were sampled at 2000 Hz from the Cybex II dynamometer and digitized with a 12-bit analog-to-digital converter (Model MP150; Biopac Systems, Inc., Goleta, CA, USA) and stored on a personal computer (Acer Aspire TC-895-UA91 Acer Inc., San Jose, CA, USA) for analysis. The torque and EMG AMP from the pre-test and post-test MVIC assessments were used to calculate Neuromuscular Efficiency (NME; normalized torque/normalized EMG AMP) [21], where the pre-test NME values were used to normalize the post-test NME values. Neuromuscular efficiency represents the level of muscle excitation required to generate a given amount of torque or force [22,23]. In addition, the torque values from the first 1 s of the RPEFT (test visit 1) were sampled from the Cybex II and used as the target torque during the TRQFT for test visit 2.

### 2.8. Statistical Analysis

In Ref. [40], a dependent *t*-test was used to examine the mean differences between anchor schemes (RPEFT vs. TRQFT) for the TTF values. The mean differences for the pre-test and post-test MVIC, EMG AMP, and NME values by anchor scheme were determined with a 2 (time: pre-test vs. post-test) × 2 (anchor scheme: RPEFT vs. TRQFT) repeated measures ANOVA. Finally, to determine the mean differences between the average values for the five item (BB, FM, HM, loss of focus, and loss of motivation) 6-point scale PTQ, the Wilcoxon Signed Rank Test [46] was utilized. For the 2 × 2 ANOVAs, significant interactions were followed-up with dependent *t*-tests. Effect size was reported as $\eta_{p}^{2}$ and Cohen’s d for ANOVAs and dependent *t*-tests, respectively. A *p*-value ≤ 0.05 was considered statistically significant and all the data were reported as mean ± SD. All calculations and statistical analyses were carried out in IBM SPSS v. 28 (Armonk, NY, USA).

## 3. Results

### 3.1. Time to Task Failure, MVIC, EMG AMP, and NME

**Figure 1:**
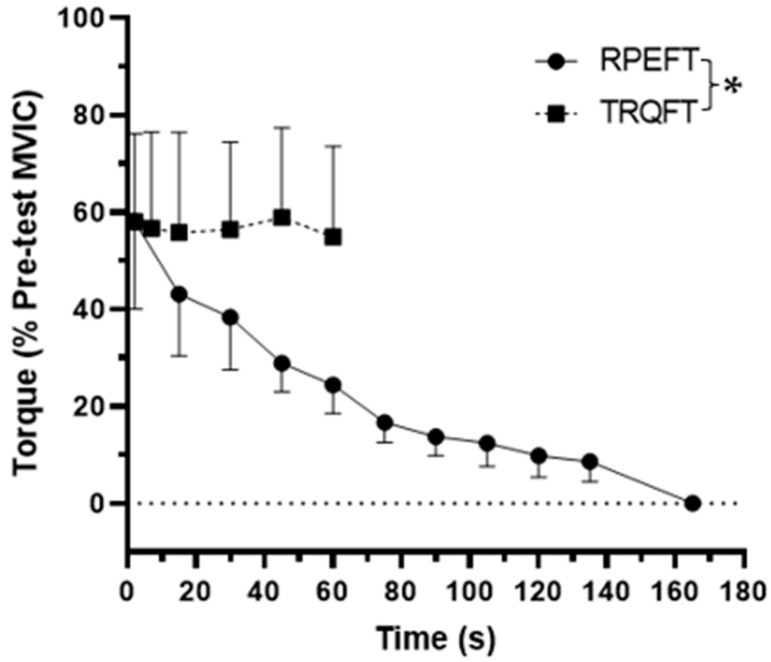
Time course of changes for the normalized (% pre-test MVIC) mean (±SD) torque values during the sustained, isometric forearm flexion tasks anchored to RPE = 8 (RPEFT) and anchored to the torque (TRQFT) produced during the first 1 s of the RPEFT. Note: * RPEFT time to task failure (174.9 ± 85.6 s) > TRQFT time to task failure (65.6 ± 68.0 s) at *p* < 0.05.

**Figure 2:**
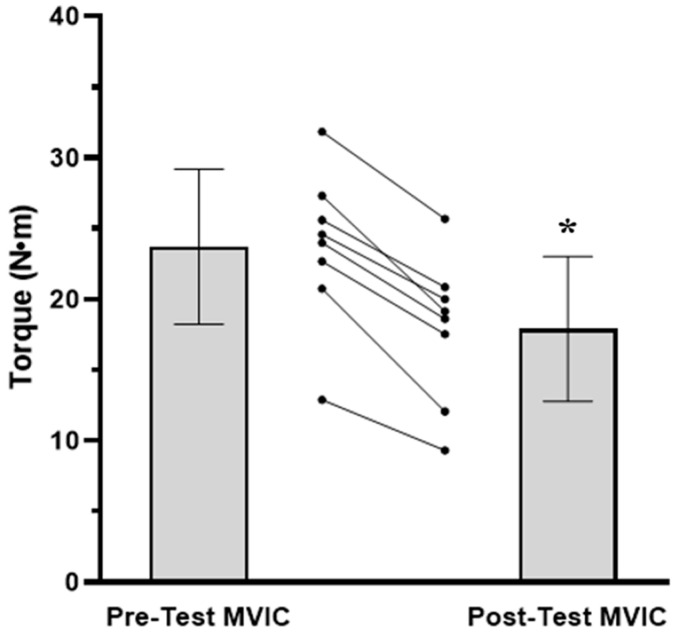
Mean (±SD) and individual maximal voluntary isometric contraction (MVIC) torque values (N·m) for the pre-test MVIC and post-test MVIC (collapsed across anchor scheme). The spaghetti graphs are the individual subject responses. Note: * pre-test MVIC > post-test MVIC at *p* < 0.05.

**Figure 3:**
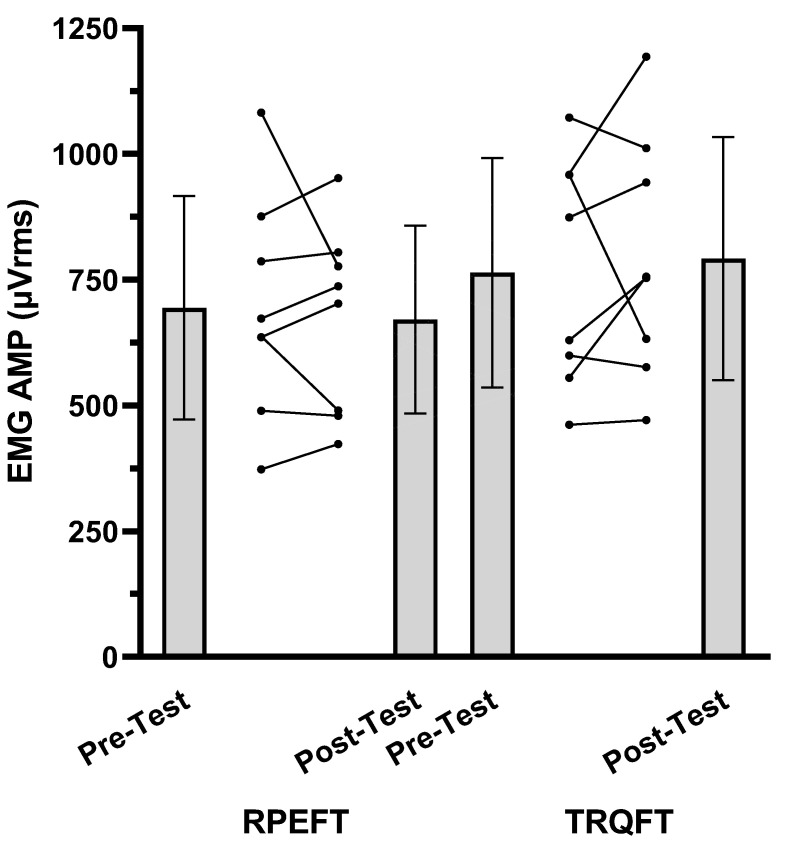
Mean (±SD) individual maximal voluntary isometric contraction (MVIC) EMG AMP (μVrms) for the pre-test MVIC and post-test MVIC assessments from test visit 1 (RPE = 8 fatigue task; RPEFT) and test visit 2 (Torque at RPE = 8 fatigue task = TRQFT). The spaghetti graphs are the individual subject responses.

**Figure 4:**
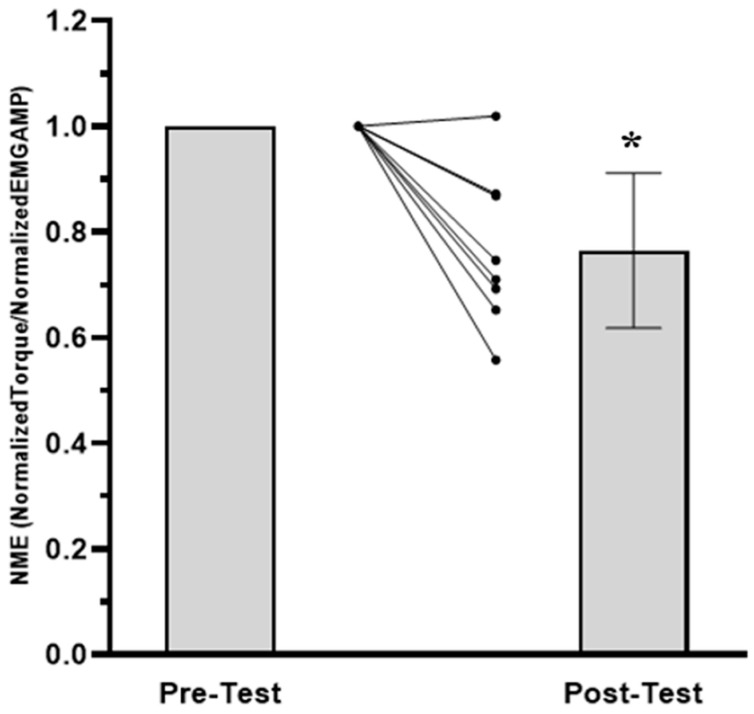
Mean (±SD) and individual neuromuscular efficiency (NME) values from the pre-test and post-test maximal voluntary isometric contraction (MVIC) assessments (collapsed across anchor scheme). Neuromuscular efficiency was defined as normalized torque/normalized EMG AMP [21]. Note: * pre-test NME > post-test NME at *p* < 0.05. The spaghetti graphs are the individual subject responses.

The results for the TTF, MVIC, EMG AMP, and NME values are presented in Figure 1, Figure 2, Figure 3 and Figure 4, respectively. For TTF, the RPEFT was significantly (*p* = 0.006, d = 1.363) greater than the TRQFT (174.9 ± 85.6 vs. 65.6 ± 68.0 s). For MVIC, there was no significant (*p* = 0.297, $\eta_{p}^{2}$ = 0.154) anchor scheme × time interaction, but there was a significant (*p* < 0.001, $\eta_{p}^{2}$ = 0.924) main effect for time (collapsed across anchor scheme) and a significant (*p* = 0.046, $\eta_{p}^{2}$ = 0.456) main effect for the anchor scheme (collapsed across time). Two separate follow-up dependent *t*-tests indicated that the pre-test MVIC values were significantly (*p* < 0.001, d = 3.261) greater than the post-test MVIC values (23.7 ± 5.5 vs. 17.9 ± 5.1 N·m), and that the pre-test MVIC values from the RPEFT visit were significantly (*p* = 0.046, d = 0.856) greater than the pre-test MVIC values from the TRQFT visit (21.9 ± 5.9 vs. 19.6 ± 4.9 N·m). For EMG AMP, there was no significant (*p* = 0.577, $\eta_{p}^{2}$ = 0.047) anchor scheme × time interaction, main effect (*p* = 0.946, $\eta_{p}^{2}$ = 0.143) for anchor scheme, or main effect (*p* = 0.173, $\eta_{p}^{2}$ = 0.248) for time. For NME, there was no significant (*p* = 0.315, $\eta_{p}^{2}$ = 0.143) anchor scheme × time interaction or main effect (*p* = 0.315, $\eta_{p}^{2}$ = 0.143) for anchor scheme (collapsed across time). There was, however, a significant (*p* = 0.003, $\eta_{p}^{2}$ = 0.746) main effect for time (collapsed across anchor scheme). The marginal mean for the pre-test NME was significantly (*p* = 0.003, d = 1.602) greater than the post-test NME (1.00 ± 0.00 vs. 0.76 ± 0.15).

### 3.2. Post-Test Questionnaire

**Figure 5:**
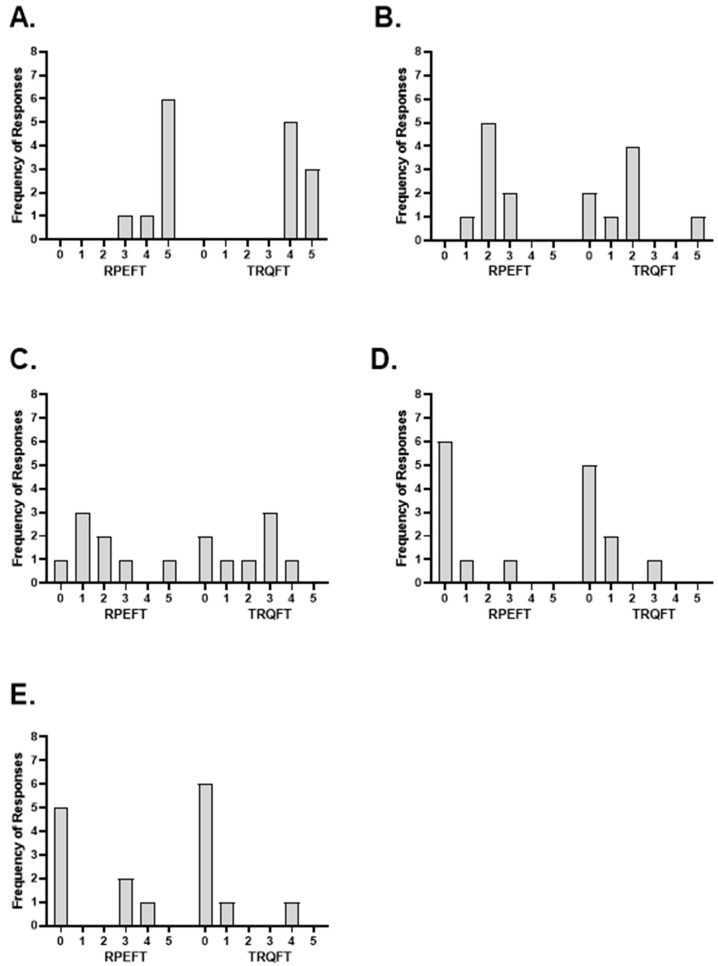
Frequency of responses for each of the five items from the Post-Test Questionnaire following the sustained, isometric forearm flexion task anchored to RPE = 8 (RPEFT) and the sustained, isometric forearm flexion task anchored to torque (TRQFT) produced during the first 1-s of the RPE = 8 task: (**A**) Biceps Brachii; (**B**) Forearm Muscles; (**C**) Hand Muscles; (**D**) Loss of Motivation; (**E**) Loss of Focus. A rating 0 indicated that the items had no contribution to the decisionto terminate the task and a rate of 5 indicated that the item had the greatest level of contribution of the five items to the decision to terminate the task. Ratings of 1–4 indicated the variying levels of intensity of contribution to the decision to terminate the task.

Five separate Wilcoxon Signed Rank Tests were used to examine the average differences between the RPEFT and TRQFT for the five-item response scores. There were no significant differences between the RPEFT and TRQFT response scores for BB fatigue (4.6 ± 0.7 vs. 4.4 ± 0.5; *Z* = −1.000, *p* = 0.317), FM fatigue (2.1 ± 0.6 vs. 1.8 ± 1.6; *Z* = −0.750, *p* = 0.453), HM fatigue (1.9 ± 1.6 vs. 2.0 ± 1.5; *Z* = −0.414, *p* = 0.679), loss of motivation (0.5 ± 1.0 vs. 0.6 ± 1.0; *Z* = −1.000, *p* = 0.317), or loss of focus (1.3 ± 1.8 vs. 0.6 ± 1.4; *Z* = −1.089, *p* = 0.276). The frequency of responses for each item is presented in Figure 5. In addition, the individual subject ratings for each item from the PTQ are provided in Table 2.

## 4. Discussion

The results indicated that the TTF for the RPEFT was 2.7 times longer than the TRQFT (174.9 ± 85.6 vs. 65.6 ± 68.0 s; Figure 1), which was due to the ability to consciously reduce the torque during the RPEFT and the requirement to maintain torque during the TRQFT. Previous studies that have utilized the RPE-Clamp model by Tucker [6] have indicated that decreases in muscle excitation (based on reductions in EMG AMP) and torque were necessary to maintain the perceptual intensity [7,12], whereas tasks anchored to a constant torque showed increased muscle excitation to sustain the target torque [47,48,49]. In theory, the reductions in muscle excitation [7,12] resulted from the conscious derecruitment of some of the activated motor units, which allowed individuals to withstand the deleterious effects of fatigue, such as metabolic perturbations, feelings of tiredness and weakness, and the inability to produce torque [2] for a longer period of time because of a reduced risk of disruption to the neuromuscular, cardiovascular, and respiratory systems [50]. The risk of systematic disruption [50], however, may be more applicable when a larger amount of muscle mass is engaged, such as during whole-body exercise or bilateral leg extensions than for unilateral forearm flexion tasks. Therefore, it is likely that the TTF for the RPEFT was longer than the TRQFT in the present study because the subjects were able to consciously derecruit motor units and reduce torque. Future research is needed to determine if the amount of engaged muscle affects tolerance to fatigue for small muscle groups because of the reduced risk to systemic homeostasis.

Unlike the present study, Keller et al. [13] reported that during the bilateral, isometric leg extensions anchored to RPE (1, 5, and 8) and force (force at RPE 1, 5, and 8), there were no differences in TTF values between anchor schemes. The results of the present study in women and those of Keller et al. [13] in men suggest that there may be muscle-, task-, and/or sex-specific differences for the effects of anchor scheme on TTF during sustained, isometric tasks. The bilateral leg extensions used in Keller et al. [13] involved greater engaged muscle mass [51,52] that produced more torque (or force) at RPE = 8 [53,54] than the unilateral forearm flexion tasks in the present study. Perhaps, these differences resulted in muscle-specific metabolic responses to fatigue related to muscle oxygenation, ATP utilization, and metabolite buildup [55,56], which influenced the effect of the anchor scheme on TTF. It is also possible, that the use of bilateral versus unilateral tasks contributed to the differences between the current findings and those of Keller et al. [13] regarding the effect of the anchor scheme on TTF. It has been hypothesized [57] that interhemispheric inhibition may be responsible for fatigue-related differences between unilateral and bilateral tasks. Furthermore, sex-differences in fatigue responses may have contributed to the differences between the current findings and those of Keller et al. [13]. For example, Hunter [58] demonstrated that women tend to be more fatigue resistant than men, which is perhaps due to a greater proportion of Type I muscle fibers, reduced metabolite accumulation due to decreased mechanical compression of blood vessels, and less feedback from group III/IV muscle afferents, which theoretically allows for greater neural excitation. Future research is needed, however, to determine if there are muscle-, task-, and/or sex-specific influences on the effects of the anchor scheme on TTF during forearm flexion and leg extension tasks.

The present findings indicated that there were no differences in the fatigue-induced decrease in MVIC for the RPEFT versus the TRQFT (Figure 2). These findings were consistent with those of Keller et al. [13] who also reported no differences in performance fatigability (13.1% decrease in force) between bilateral, isometric leg extension tasks anchored to RPE and force. Gandevia [59] suggested a hypothetical construct of fatigue termed the “sensory” tolerance limit (STL), which suggested that exercise performance may deteriorate “…because the consequences of continuing the task become sufficiently unattractive” (p. 1766). Furthermore, Hureau et al. [25] conceptualized the STL as a global model of fatigue where the sum of all the feedback from systems directly and/or indirectly involved with the exercise task, as well as the feedforward corollary discharge associated with central motor command determine the intensity of exercise that can be maintained at a tolerable level. Task failure occurs when the individual reaches a finite level of stimulation (i.e., their STL) from all feedback and feedforward sources. Based on the STL [25,59], the task is terminated when the individual interprets the exercise as “…sufficiently unattractive…” [25] to continue, or the individual can choose to decrease the exercise intensity so that the task can be continued. In theory, when a submaximal task is anchored by torque, the fatigue responses are characterized by a combination of increases in the central drive and additional motor unit recruitment to compensate for fatigued motor units, corollary discharges, and sensory afferent feedback that progresses until the STL is reached [25,37]. When a task is anchored to RPE, however, the STL is consciously set at a predetermined perception of exertion [6]. Thus, during the RPEFT, the torque was consciously decreased [7,8,59] to maintain the prescribed RPE, avoid the STL, and sustain the task. Therefore, it may be that, in the present study, both the RPEFT and TRQFT were discontinued when the individuals reached their STL, which took longer during the RPEFT due to the ability to reduce torque. The comparable pre-test to post-test decreases in MVIC for the two anchor schemes may have reflected reaching the same STL, even though the TTF values differed. Furthermore, the relative contributions from central and peripheral mechanisms to reach the STL may have differed between the anchor schemes, but ultimately led to the same magnitude of fatigue-induced decreases in MVIC [60,61]. In addition, the contrast in the findings for TTF, but the similar decreases in the pre- to post-test MVIC values between the present study and those of Keller et al. [13], may suggest that the STL is manifested differently during bilateral versus unilateral tasks. Future research is recommended to determine if this is the case.

These results indicated that there were no fatigue-induced changes in EMG AMP (Figure 3) following the RPEFT (1.1%) or TRQFT (−5.8%), but there were fatigue-induced decreases in NME (23%; Figure 4), regardless of the anchor scheme. Neuromuscular parameters such as EMG AMP and NME have been used to make inferences regarding fatigue-induced contributions from ntral and/or peripheral mechanisms to performance fatigability following sustained, isometric forearm flexion tasks [10,22,62]. For example, Miller et al. [22] reported 90% and 40% decreases in force and NME, respectively, during a sustained, isometric task of the adductor pollicis muscle anchored to 50% MVIC and suggested that the impairment of NME was due to a buildup of metabolites within the intramuscular milieu that likely contributed to the decline in force output. Previous studies [8,9,10] have hypothesized that sustained, isometric tasks anchored to a high perceptual intensity may result in reduced blood flow and the occlusion of vascular beds as a consequence of intramuscular pressure [63], which may cause peripheral fatigue due to the buildup of metabolic byproducts such as inorganic phosphate (Pi), calcium ions (Ca^2+^), extracellular potassium (K^+^), and magnesium (Mg) [55,64,65,66]. Furthermore, peripheral fatigue can impair excitation–contraction coupling through the effects of intramuscular metabolic perturbations on sarcoplasmic reticulum calcium release and re-uptake kinetics, calcium sensitivity for binding with troponin, and cross-bridge cycling [64,65,67]. Alternatively, central fatigue may occur due to buildup of hydrogen ions (H^+^) in the interstitial space [68] that cause inhibitory feedback from group III/IV muscle afferent fibers to the motor areas of the brain and leads to decreases in the central motor command, force production, and synaptic nerve responsiveness via supraspinal and spinal mechanisms [60,68]. It has also been suggested [60] that central fatigue is characterized by a decrease in the motor unit firing rate due to a decrease in the excitatory input and an increase in the inhibitory input, as well as a decrease in the motor neuron excitability. Although the use of EMG AMP to make inferences regarding the central motor command has been questioned [14,48], there were no mean fatigue-induced changes in EMG AMP following the RPEFT and TRQFT in the present study, which may have reflected no changes in the muscle excitation associated with central fatigue. Therefore, the magnitude of performance fatigability reported in the present study may have been due to peripheral fatigue that resulted in excitation–contraction coupling failure, as evidenced by decreased NME. In addition, the mean power frequency from the mechanomyographic signal (MMG MPF), which qualitatively reflects changes in the global firing rate of activated, unfused motor units [69], as well as the interpolated twitch and resting potentiated twitch amplitude techniques, may improve differentiating the central and peripheral contributions to MVIC loss. Future research should use the interpolated twitch and potentiated twitch amplitude techniques along with neuromuscular responses to better determine the contributions of central and peripheral fatigue to MVIC loss following sustained tasks that are anchored to a constant RPE and torque.

The five PTQ items in the present study were designed to assess the locations and magnitudes of perceived sensations that potentially contributed to reaching the STL and, therefore, terminating the fatiguing tasks (Table 2 and Figure 5). The perceived sensations from the BB, FM, and HM likely reflected the localized, peripheral sensory feedback via group III/IV afferent neurons to the supplementary motor area [70], possibly due to exertion, pain, discomfort, or strain [3,5,31,39] caused by restricted blood flow and intramuscular metabolic perturbations during the fatiguing tasks [2,3]. It is possible that the loss of motivation to continue the tasks also resulted from the same peripheral sensations and feedback mechanisms as the BB, FM, and HM, or from central factors such as boredom or executive brain function, which involved feedforward mechanisms from the anterior cingulate cortex and dorsolateral prefrontal cortex [39,71]. The contribution to task termination from the loss of focus likely involved feedforward mechanisms from the anterior inferior parietal lobe of the brain [72] due to centrally mediated factors such as attentional shift, which can be produced in a goal-directed manner (i.e., voluntary allocation of attention) or due to stimulus-driven processing (i.e., involuntary allocation of attention) [39,73]. Although there were no mean differences between the RPEFT and TRQFT for any of the PTQ items, there were a number of interindividual differences in the responses (Table 2 and Figure 5). Eighty-one percent of the ratings (13 out of 16) indicated that sensations from the BB were the primary contributors to the decision to terminate the tasks, while 69% (11 out of 16) also indicated some level of contribution from the FM and HM (Table 2 and Figure 5). Except for subject seven for both anchor schemes and subject five for the TRQFT (Table 2), all ratings from the BB were higher than any of the other PTQ items. These findings suggested that the peripheral sensory feedback was predominant, but not the only mechanism that contributed to the decision to terminate the task. Furthermore, 31% (10 out of 32) of the subjects’ ratings indicated that a loss of focus and a loss of motivation contributed to the decision to terminate the task (Table 2; Figure 5D,E, respectively). Previously, Rejeski [38] hypothesized that during low-to-moderate intensity exercise, psychological factors exert a greater influence on perceived exertion than neuromuscular or physiological factors. At higher intensities, however, neuromuscular and physiological factors override the influence of the psychological factors [38]. In addition, Hutchinson and Tenenbaum [39] suggested that during low intensity exercise, focus can easily shift between internal and external stimuli, but during high intensity or maximal exercise, focus cannot be voluntarily controlled “…but is compelled to remain internal and narrow due to the overwhelming salience of the effort-related sensations” (p. 166). The current findings, however, indicated that for three of the eight subjects (31%), a loss of motivation and/or loss of focus contributed to their decision to terminate the tasks with ratings that ranged from 1–4 (Table 2). Five of the subjects, however, selected ratings of zero for both loss of focus and loss of motivation. Thus, in addition to the feedback mechanisms associated with sensations from the BB, FM, and HM, for a minority of subjects, the peripheral sensory feedback associated with motivation and/or centrally mediated feedforward mechanisms from focus and motivation contributed to the decision to terminate the tasks.

A potential limitation of the present study was the inability of the sample size to detect significant mean differences for EMG AMP. Furthermore, it has been suggested [74] that exercise experience can mediate the relationship between task performance, RPE, and perceived fatigability. Thus, it may be that a lack of experience with the isometric, forearm flexion task could have influenced the subjects’ abilities to determine their RPE during the RPEFT and may have affected the subjects’ abilities to sustain torque during the TRQFT. Another limitation of the present study was the normalization procedures used to determine NME, as it has been previously suggested [75] to potentially result in an inappropriate interpretation of fatigue-induced changes in peripheral mechanisms. Furthermore, the menstrual cycle and/or use of oral contraceptives were not considered. Previous studies [76,77] have reported that RPE may be influenced by different phases of the menstrual cycle, as well as the timing of oral contraceptive use. Given this information, the processing of RPE during the RPEFT in the present study may have been influenced depending on the phase of the menstrual cycle and the timing of oral contraceptive use. Concerning the PTQ, loss of motivation and loss of focus may be mediated by a combination of feedback and feedforward processes that include peripheral and central factors [2,24,39,71]. Future studies should separate these items into multiple components to identify with greater specificity the potential contributions to the decision to terminate a task anchored to RPE and torque. For example, loss of motivation could be separated into boredom, potential motivation (i.e., the maximal effort one is willing to exert), and other motivational variables such as the incentive value of the anticipated outcome and the desire to participate, while loss of focus could be separated into external attention shift and internal attention shift.

In summary, the present study examined the influence of an anchor scheme (RPEFT vs. TRQFT) on changes in performance fatigability and neuromuscular responses, as well as PTQ responses that represented factors associated with perceived fatigability that contributed to the decision to terminate the task. The present findings indicated that the RPEFT had a longer TTF versus the TRQFT, which was likely due to the ability to consciously derecruit motor units and reduce torque. Furthermore, there were similar fatigue-induced decreases in MVIC following the RPEFT and TRQFT, which may have indicated that subjects reached the same individual STL for both tasks. In addition, given the lack of changes in EMG AMP following the RPEFT and TRQFT, we postulated that this may have reflected no change in muscle excitation associated with central fatigue and that it was due to peripheral fatigue that resulted in excitation–contraction coupling failure as evidenced by decreased NME. Finally, although the PTQ responses indicated no mean differences between the anchor schemes, there were substantial interindividual differences in the responses. It was hypothesized that the perceived sensations from the BB, FM, and HM likely reflected peripheral sensory feedback via group III/IV afferent neurons. For loss of motivation and loss of focus, we hypothesized that these items were likely mediated by a single or a combination of peripheral and central factors involved with feedback and/or feedforward processes in higher brain centers. The present findings provide a foundation for future studies to assess the contributing performance-related factors and psychological factors to the decision to terminate the task. Additional studies should assess other psychological factors, such as overall perceived fatigue, affective valence, and pain perception, to determine if they contribute to the decision to terminate a task anchored to RPE or torque. Future studies should also utilize the interpolated twitch and potentiated twitch amplitude techniques, along with MMG MPF, and, potentially, decomposition techniques to examine the relative contributions of central and peripheral mechanisms to MVIC loss following sustained tasks anchored to a constant RPE and torque.

## Figures and Tables

**Table 1:** Time course of procedures.

Orientation Session Informed consent. Health History Questionnaire. Age, height, and body mass recorded. Read standardized anchoring instructions (OMNI-RES scale). Standardized warm-up: 4 repetitions of 3 s submaximal (50% to 75% of maximal effort) isometric forearm flexion muscle actions. 2 repetitions of 3 s isometric forearm flexion MVICs to set perceptual anchor of RPE = 10. Brief (~1 min) sustained isometric task anchored to RPE = 8 at an elbow joint angle of 125°.	Test Visit 1 Standardized warm-up. Read standardized anchoring instructions (OMNI-RES scale). Pre-test: 2 repetitions of 3 s MVICs at an elbow joint angle of 125°. Sustained, isometric forearm flexion task anchored to RPE = 8 (OMNI-RES scale) performed at an elbow joint angle of 125° to task failure. Post-test: 2 repetitions of 3 s MVICs at an elbow joint angle of 125°. Post-test questionnaire.	Test Visit 2 Standardized warm-up. Pre-test: 2 repetitions of 3 s MVICs at an elbow joint angle of 125°. Sustained, isometric forearm flexion task anchored to torque corresponding to torque produced during RPE = 8 (OMNI-RES scale) task, performed at an elbow joint angle of 125° to task failure. Post-test: 2 repetitions of 3 s MVICs at an elbow joint angle of 125°. Post-test questionnaire.

MVIC = maximal voluntary isometric contraction; RPE = ratings of perceived exertion.

**Table 2:** Individual subject ratings for each item from the Post-Test Questionnaire following the sustained, isometric tasks from test visit 1 (RPEFT) and test visit 2 (TRQFT).

Subjects	RPEFT					TRQFT				
	Biceps Brachii	Forearm Muscles	Hand Muscles	Loss of Motivation	Loss of Focus	Biceps Brachii	Forearm Muscles	Hand Muscles	Loss of Motivation	Loss of Focus
1	5	2	2	0	0	5	1	1	0	0
2	5	2	1	0	0	5	2	0	0	0
3	5	2	3	0	0	4	2	3	0	0
4	5	3	2	0	0	4	2	2	0	0
5	5	3	1	1	4	4	5	3	1	1
6	4	2	0	0	0	4	0	0	0	0
7	3	1	5	3	3	4	2	4	3	4
8	5	2	1	0	3	5	0	3	1	0

Note: The five items (biceps brachii, forearm muscles, hand muscles, loss of focus, and loss of motivation) were rated on a 6-point (0–5) scale. A rating of 0 indicated that the item had no contribution to the decision to terminate the task and a 5 indicated that the item had the greatest level of contribution to the decision to terminate the task. Ratings of 1–4 indicated the varying levels of intensity of contribution to the decision to terminate the task. RPEFT = RPE fatigue task; TRQFT = torque fatigue task.

## Data Availability

The data sets generated during and/or analyzed during the present study are available from the corresponding author upon reasonable request.

## Appendix A

### Standardized OMNI-RES Instructions

You will be asked to set an anchor point for both the lowest and highest values on the perceived exertion scale. In order to set the lowest anchor, you will be asked to lay quietly without contracting your forearm flexor muscles to familiarize yourself with a zero. Following this, you will be asked to perform a maximal voluntary isometric contraction to familiarize yourself with a 10. When instructed to match a perceptual value corresponding to the OMNI-RES scale, perceived exertion should be relative to these defined anchors.

## Appendix B

Five-item, 6-point (0–5) Post-Test Questionnaire.

Please rate each item from 0–5 where a 0 indicates that the item had no contribution to the decision to terminate the task and a 5 indicates that the item had the greatest level of contribution to the decision to terminate the task. If you feel that the item’s intensity of contribution to terminating the task is somewhere in between these two reference points, please rate it accordingly with a 1–4 rating. Finally, if you feel that more than one item contributed equally to the decision to terminate the task, rate them the same.						
	0 No Contribution	1	2	3	4	5 Greatest level of Contribution
Biceps Brachii	0	1	2	3	4	5
Forearm Muscles	0	1	2	3	4	5
Hand Muscles	0	1	2	3	4	5
Loss of Mental Focus	0	1	2	3	4	5
Loss of Motivation	0	1	2	3	4	5

### Instructions for the Post-Test Questionnaire

This is the post-test questionnaire. It contains five separate items: biceps brachii, forearm muscles, hand muscles, loss of focus, and loss of motivation. In this context, the biceps brachii, forearm muscles, and hand muscles are simply to help you identify the potential location of sensations such as strain, discomfort, and/or pain that occurred during the task. Furthermore, loss of motivation refers to a state of mind where the task, for whatever reason, became sufficiently unattractive and you decided to terminate the task. Finally, loss of focus refers to a shift in attention to other tasks or responsibilities, which may have caused a disruption in the ability to maintain the prescribed RPE or torque value. Please rate each item from 0–5 where a 0 indicates that the item had no contribution to the decision to terminate the task and a 5 indicates that the item had the greatest level of contribution to the decision to terminate the task. If you feel that the item’s intensity of contribution to terminating the task is somewhere in between these two reference points, please rate it accordingly with a 1–4 rating. Finally, if you feel that more than one item contributed equally to the decision to terminate the task, rate them the same.
